# Supplementary material for: From sequence to enzyme mechanism using multi-label machine learning
Source: BMC Bioinformatics. 2014 May 19;15:150. doi: 10.1186/1471-2105-15-150 (PMC4229970; doi:10.1186/1471-2105-15-150)
Supplement: Additional file 2 — Java code of ml2db. Additional file ml2db_code.tar.gz contains the Java source code to run the multi-label machine learning experiments and save the results to database. The code’s Javadoc is included. [file 1471-2105-15-150-S2.zip › additional file 2/ml2db/ecmulan/doc/uk/ac/ed/inf/mulanxml/test/LocalDbReaderTest.html]

LocalDbReaderTest


JavaScript is disabled on your browser.


- Overview
- Package
- Class
- Use
- Tree
- Deprecated
- Index
- Help

- Prev Class
- Next Class

- Frames
- No Frames

- All Classes

- Summary:
- Nested |
- Field |
- Constr |
- Method

- Detail:
- Field |
- Constr |
- Method


uk.ac.ed.inf.mulanxml.test

## Class LocalDbReaderTest

- java.lang.Object
- - junit.framework.Assert
  - - junit.framework.TestCase
    - - uk.ac.ed.inf.mulanxml.test.LocalDbReaderTest

- All Implemented Interfaces:
  :   junit.framework.Test

  ---

    

  ```
  public class LocalDbReaderTest
  extends junit.framework.TestCase
  ```

  Class

  Version:
  :   5 May 2010

  Author:
  :   Luna De Ferrari luna.deferrari-at-ed.ac.uk

- - ### Field Summary

    Fields

    | Modifier and Type | Field and Description |
    | --- | --- |
    | `static java.lang.String` | `EC_FIELD` |
    | `static java.lang.String` | `GET_EC_QUERY_1` |
    | `static java.lang.String` | `GET_EC_QUERY_2` |
    | `static java.lang.String` | `STATUS_FIELD` |
    | `static java.lang.String` | `TEST_DB_CONN_PATH` the test database |
  - ### Constructor Summary

    Constructors

    Constructor and Description

    `LocalDbReaderTest()`
  - ### Method Summary

    Methods

    | Modifier and Type | Method and Description |
    | --- | --- |
    | `void` | `createTestTable()` Create a test EC table in the test database |
    | `void` | `tearDown()` |
    | `void` | `testGetEcList()` |

    - ### Methods inherited from class junit.framework.TestCase

      `countTestCases, getName, run, run, runBare, setName, toString`
    - ### Methods inherited from class junit.framework.Assert

      `assertEquals, assertEquals, assertEquals, assertEquals, assertEquals, assertEquals, assertEquals, assertEquals, assertEquals, assertEquals, assertEquals, assertEquals, assertEquals, assertEquals, assertEquals, assertEquals, assertEquals, assertEquals, assertEquals, assertEquals, assertFalse, assertFalse, assertNotNull, assertNotNull, assertNotSame, assertNotSame, assertNull, assertNull, assertSame, assertSame, assertTrue, assertTrue, fail, fail, failNotEquals, failNotSame, failSame, format`
    - ### Methods inherited from class java.lang.Object

      `equals, getClass, hashCode, notify, notifyAll, wait, wait, wait`

- - ### Field Detail


    - #### GET\_EC\_QUERY\_1

      ```
      public static final java.lang.String GET_EC_QUERY_1
      ```

      See Also:
      :   Constant Field Values


    - #### GET\_EC\_QUERY\_2

      ```
      public static final java.lang.String GET_EC_QUERY_2
      ```

      See Also:
      :   Constant Field Values


    - #### TEST\_DB\_CONN\_PATH

      ```
      public static final java.lang.String TEST_DB_CONN_PATH
      ```

      the test database

      See Also:
      :   Constant Field Values


    - #### EC\_FIELD

      ```
      public static java.lang.String EC_FIELD
      ```


    - #### STATUS\_FIELD

      ```
      public static java.lang.String STATUS_FIELD
      ```
  - ### Constructor Detail


    - #### LocalDbReaderTest

      ```
      public LocalDbReaderTest()
      ```
  - ### Method Detail


    - #### createTestTable

      ```
      public void createTestTable()
      ```

      Create a test EC table in the test database


    - #### tearDown

      ```
      public void tearDown()
                    throws java.lang.Exception
      ```

      **Overrides:**
      :   `tearDown` in class `junit.framework.TestCase`

      Throws:
      :   `java.lang.Exception`


    - #### testGetEcList

      ```
      public void testGetEcList()
      ```


- Overview
- Package
- Class
- Use
- Tree
- Deprecated
- Index
- Help

- Prev Class
- Next Class

- Frames
- No Frames

- All Classes

- Summary:
- Nested |
- Field |
- Constr |
- Method

- Detail:
- Field |
- Constr |
- Method
